# ST-Elevation Myocardial Infarction in Situs Inversus Dextrocardia: A Case Report

**DOI:** 10.7603/s40602-016-0010-7

**Published:** 2016-12-06

**Authors:** Koh Keng Tat, Asri Said, Oon Yen Yee, Siti Nadia binti Che Adinan, Ong Tiong Kiam

**Affiliations:** 1 Department of Cardiology, Sarawak Heart Centre, 94000 Kota Samarahan, Sarawak Malaysia; 2 Faculty of Medicine and Health Sciences,, University Malaysia Sarawak, Kota Samarahan, Sarawak Malaysia

**Keywords:** dextrocardia, situs inversus, STEMI, acute myocardial infarction, percutaneous coronary intervention

## Abstract

ST-elevation myocardial infarction (STEMI) in situs inversus dextrocardia is a rare combination and poses many challenges in terms of diagnosis and management. These include the early detection of dextrocardia as well as the interpretation of the ECG. In addition, percutaneous coronary intervention could be challenging in the setting of dextrocardia because of diffi culty in cannulating the coronary arteries, selection of catheters, catheter manipulation, image acquisition and interpretation.

## Introduction

Situs inversus dextrocardia is a rare congenital anomaly that produces a “mirror image” of the heart and viscera. The incidence is estimated to be around 0.1 to 0.2/1000 population^1^. Affected individuals usually have a structurally normal heart but may pose diagnostic and management challenges in acute coronary syndrome.

## Case report

**Figure 1:**
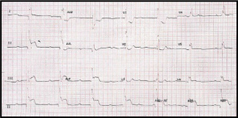

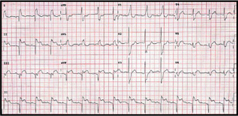

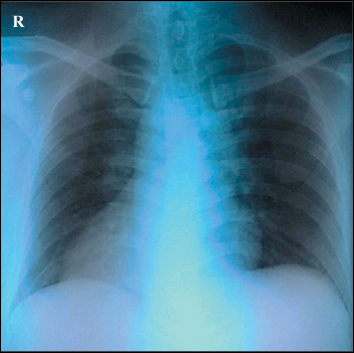
Figure 1.

A 45-year-old gentleman presented with sudden onset of central chest pain associated with diaphoresis and shortness of breath. He was an active smoker (>30 pack years) with a past medical history of Type 2 diabetes mellitus and bronchial asthma. He arrived at the emergency department of a district hospital (in Sarawak) within 2 hours from the onset of chest pain. His blood pressure at presentation was 84/48mmHg, heart rate 43bpm, afebrile, oxygen saturation (SaO_2_) 100% under room air and pain score 10/10. His left sided ECG showed complete heart block with ST elevation in leads II, III, aVF and ST depression in V1 and V2. (Figure 1A) Chest X-ray was reported as a ‘rotated film’. (Figure 1C) A diagnosis of inferoposterior ST-elevation myocardial infarction (STEMI) was made. He was given T. aspirin 300mg, T. clopidogrel 300mg, T. simvastatin 40mg, subcutaneous enoxaparin 60mg and IV tenecteplase 35mg (body weight 69kg). IV fentanyl 50mcg and IV morphine 3mg were also given.

One hour after thrombolysis, his pain score reduced to 5/10. Blood pressure was 133/94mmHg (no inotropes), heart rate 75bpm, and SaO2 of 100%. Left sided ECG showed resolution of complete heart block but with <50% resolution of ST elevation in leads II, III and aVF. He was immediately transferred to our cardiology centre for rescue percutaneous coronary intervention (PCI).

He arrived 3 hours later (340km by road). His blood result for Creatinine Kinase Myocardial Band (CK-MB) was 273.5ng/ml, Creatinine Kinase >4267IU/ml and serum creatinine 87umol/L. He was immediately transferred to the invasive cardiac laboratory for rescue PCI.

***Figure 1A:***Standard left sided ECG showing complete heart block, RBBB, ST elevation in II, III, aVF and ST depression in V1 and V2. Note the reversed polarity of both atrial and ventricular.

***Figure 1B:*** Right sided ECG before PCI: resolution of complete heart block but incomplete resolution of ST elevation in II, III, aVF. Note that the atrial polarity is normalized but the ventricular polarity is still reversed (only the precordial leads were reversed but the limb leads in standard position)

***Figure 1C:*** Chest x-ray revealed right sided cardiac silhouette, right sided aortic knuckle and inverted bronchial situs.

Coronary angiography was done via a right trans-radial approach using a 5F Optitorque^TM^ Tiger diagnostic catheter (Terumo IS, Somerset, US). While passing the catheter to the aorta, the operator noticed that the patient had dextrocardia from the cardiac silhouette under fl uoroscopy. The diagnostic catheter was unable to engage both the left coronary artery (LCA) and right coronary artery (RCA). Decision was made to convert to trans-femoral approach using 5F Judgkins Left 4cm (JL4) and 5F Judgkins Right 4cm (JR4) catheters. The JL4 catheter passed through the aorta, which was on the right side of the spine and cannulated the left coronary artery with ease. The JR4 catheter cannulated the right coronary artery with a counterclockwise torque (instead of the usual clockwise torque). The coronary angiogram showed that the morphological left coronary artery was normal. There was an ulcerated plaque in the proximal RCA and thrombus in the distal RCA, posteriolateral ventricular (PLV) branch and posterior descending artery (PDA) branch.

**Figure 2:**
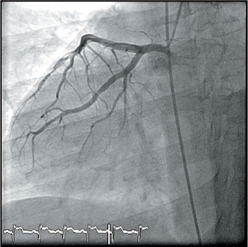

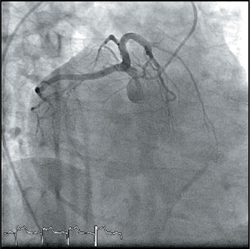

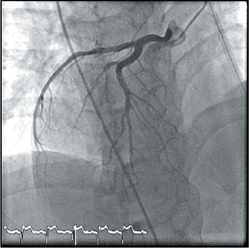

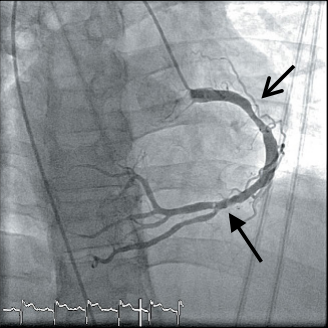
Figure 2.

Modifi cations were made for image acquisition during coronary angiography (Figure 2). The morphological LCA was engaged in the usual AP projection, but the morphological RCA was engaged in RAO projection (instead of LAO). For the LCA, LAO Caudal view became the ‘spider’ view; RAO Caudal became the LAO Caudal view and RAO Cranial became the LAO Cranial View. For the RCA, LAO became the RAO view and LAO became the RAO view.

***Figure 2A:*** Left coronary artery: LAO Caudal

***Figure 2B:*** Left coronary artery: RAO Caudal

***Figure 2C:*** Left coronary artery: RAO Cranial

***Figure 2D:*** Right coronary artery: RAO.

***Arrow:*** ulcerated plague at proximal RCA.

***Arrow head:*** thrombus at distal RCA, PLV and PDA branches.

As the patient had TIMI 3 fl ow in the RCA, we decided not to PCI the proximal RCA lesion. IV tirofi ban was started to treat the distal thrombus and the patient was observed in the coronary care unit. Further history taking from the patient revealed that he was married for 10 years without children. Bedside abdominal ultrasonography showed inversion of the abdominal viscera: liver and gallbladder were on the right and spleen on the left. The patient had a history of bronchial asthma but no recurrent sinusitis or bronchiectasis. In summary, he had situs inversus which was undiagnosed prior to this STEMI event.

The patient was discharged well after 4 days with an appointment for outpatient cardiac rehabilitation and intensive cardiovascular risk factors modifi cation.

## Discussion

STEMI in situs inversus dextrocardia is a rare combination. Literature search in the PubMed found fewer than 70 similar case reports. To the best of our knowledge, this is the fi rst case report in Malaysia

The ECG interpretation for STEMI can be deceiving if dextrocardia is not detected early. In our case, the chest x-ray showed a right-sided cardiac silhouette, right-sided aortic knuckle and inverted bronchial situs (gastric bubble not well seen in the chest x-ray) (Figure IC). The standard left sided 12-lead ECG showed typical reversal of atrial and ventricular polarity with negative p waves in I and aVL and reversed R wave progression in precordial leads (figure 1A). Fortunately, this patient had an inferoposterior STEMI and not an anterior STEMI, which might have resulted in a missed diagnosis if a right sided ECG was not done^2^.

Performing coronary angiography and PCI in patients with dextrocardia and situs inversus can be a challenge to the operator. Majority of case reports favoured trans-femoral over trans-radial access^3^. In the previous case report of trans-radial multivessel PCI in patient with situs inversus dextrocardia, engagement of LCA ostium using 5F Tiger catheter was diffi cult and had to be changed to Judgkins Left 4cm^4^. In our case, trans-radial engagement of both LCA and RCA were unsuccessful. In our opinion, urgent PCI in dextrocardia should be done via a trans-femoral approach to avoid unnecessary delay.

The double-inversion technique developed by Goel PK utilizing the “horizontal sweep reversal” feature of the fl uoroscopy may help in coronary artery image acquisition and interpretation^5^. In our case, image acquisition and interpretation were possible with right-left mirror image reversal as described above. In the previous case report of PCI in dextrocardia using double-inversion technique, there was an artifi cial reversal of visualization of responses of catheters and coronary wires to normal manipulation^3^.

In conclusion, situs inversus dextrocardia can present with STEMI and this condition should be identifi ed early as it infl uences the interpretation of ECG and subsequent management. Coronary angiography and PCI in patient with dextrocardia pose important challenges to the operators. These challenges include arterial access, selection of catheters, techniques of torque of catheter and lastly image acquisition and interpretation.
